# At the Intersection of Biomaterials and Gene Therapy: Progress in Non-viral Delivery of Nucleic Acids

**DOI:** 10.3389/fbioe.2019.00131

**Published:** 2019-06-04

**Authors:** Hasan Uludag, Anyeld Ubeda, Aysha Ansari

**Affiliations:** ^1^Department of Chemical and Materinals Engineering, University of Alberta, Edmonton, AB, Canada; ^2^Department of Biomedical Engineering, University of Alberta, Edmonton, AB, Canada; ^3^Faculty of Pharmacy and Pharmaceutical Sciences, University of Alberta, Edmonton, AB, Canada

**Keywords:** biomaterials, gene medicine, nucleic acid delivery, nanoparticle, siRNA, mRNA, pDNA delivery, T-cell therapy

## Abstract

Biomaterials play a critical role in technologies intended to deliver therapeutic agents in clinical settings. Recent explosion of our understanding of how cells utilize nucleic acids has garnered excitement to develop a range of older (e.g., antisense oligonucleotides, plasmid DNA and transposons) and emerging (e.g., short interfering RNA, messenger RNA and non-coding RNAs) nucleic acid agents for therapy of a wide range of diseases. This review will summarize biomaterials-centered advances to undertake effective utilization of nucleic acids for therapeutic purposes. We first review various types of nucleic acids and their unique abilities to deliver a range of clinical outcomes. Using recent advances in T-cell based therapy as a case in point, we summarize various possibilities for utilizing biomaterials to make an impact in this exciting therapeutic intervention technology, with the belief that this modality will serve as a therapeutic paradigm for other types of cellular therapies in the near future. We subsequently focus on contributions of biomaterials in emerging nucleic acid technologies, specifically focusing on the design of intelligent nanoparticles, deployment of mRNA as an alternative to plasmid DNA, long-acting (integrating) expression systems, and *in vitro*/*in vivo* expansion of engineered T-cells. We articulate the role of biomaterials in these emerging nucleic acid technologies in order to enhance the clinical impact of nucleic acids in the near future.

## Introduction

Synthetic and naturally derived biomaterials have been firmly entrenched in technologies intended to deliver therapeutic and diagnostic agents in a clinical setting. Biomaterials typically package the agents in a form that effectively deliver them to desired sites of actions without being impeded by physiological clearance mechanisms. They could additionally provide stability to agents in the physiological milieu as well as incorporate elements that can respond to physiological stimuli to enhance the functionality of delivered agents. Their ability to incorporate therapeutic and diagnostic agents is nowhere more demanding than the attempts to employ nucleic acid-based agents, so called *gene medicines*. Nucleic acids provide seemingly infinite opportunities to undertake molecular therapy and remedy the abnormal physiology, and furthermore “personalize” the intervention with the knowledge of patient-specific complementary information. Ever since the recombinant technology has been advanced to produce natural and engineered proteins en mass (Gräslund et al., 2008), various possibilities with gene medicines have excited clinicians that are eager to replace the conventional small molecular drugs that are prone to non-specific effects on a multitude of cellular systems, and face resistance once the innate physiological mechanisms are induced by the rogue cells in order to overcome the drug effects. The recent explosion of molecular understanding of the participation of deoxyribonucleic acid (DNA) and ribonucleic acid (RNA) biomolecules in control of the cell physiology has further garnered excitement in the field to commercialize a range of older nucleic acids (e.g., antisense oligonucleotides, plasmid DNA, etc.) and emerging agents such as short interfering RNA (siRNA), non-coding microRNAs (miRs), and messenger RNA (mRNA). The latter has particularly garnered exuberant interest from the business community with the largest biotech US IPO of 2018 being undertaken by Moderna Therapeutics that focusses on development of mRNA therapeutics.

In this review article, we will summarize recent exciting developments in gene medicines, knowledge gaps in the literature and outline future avenues of fruitful activity that will enable biomaterials to “propel” DNA and RNA based agents into the clinical realm. The range of promising nucleic acids is initially summarized providing the reader with a glimpse of clinical possibilities with them. The potential impact of different nucleic acids is presented and their perceived advantages and limitations are summarized when deployed in a clinical setting. Using the T-cell therapy paradigm, we will explore the possible impact of biomaterials in implementing this mode of therapy as a representative case for an emerging, broad-impact technology. We anticipate similar technology platforms based on *ex vivo* modified/expanded cells to find clinical validation in the treatment of an increasing number of diseases. Finally, we articulate emerging areas in nucleic acid therapeutics that will be impacted by employment of biomaterials, concentrating on intelligent nanoparticles (NPs), *ex vivo* cell expansion, mRNA delivery, and long-term transgene expression. This review will primarily focus on (i) therapeutic (rather than diagnostic) modalities, and (ii) non-viral, biomaterials-centered methods to undertake effective delivery of nucleic acids. The authors acknowledge that exciting developments are taking place in viral design and engineering to undertake clinical therapy, but we refer the reader to other sources on recent developments on this front (Schott et al., 2016; Lundstrom, 2018).

## Spectrum of Nucleic Acids for Clinical Utility

**Figure 1:**
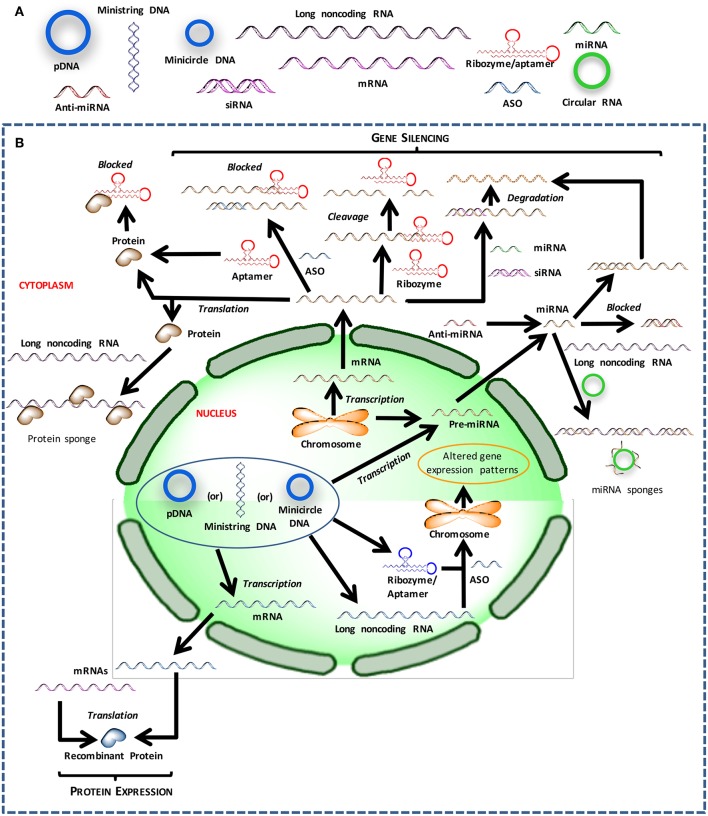
Different nucleic acids that could be used to derive therapeutic outcomes. **(A)** Major types of nucleic acids used to modulate cell behavior and could serve as therapeutic agents. **(B)** Intracellular trafficking and site of action for intervention with different types of nucleic acids.

The crux of gene medicine relies on the ability of nucleic acids to alter the physiology of a target cell. It is critical to understand the properties and physiological functions of different nucleic acids, especially at their site of action, to select the appropriate biomaterials carrier for effective transfection (Figure 1). The transient nature of the functional effects achieved with most nucleic acids forces the practitioners to choose the right target for an effective therapy. Targets whose silencing temporarily halts or simply slows down the pathological changes will not be desirable; oncogenes whose silencing lead to irreversible processes such as apoptosis induction, or targets that can sensitize the cells to deadly drug action subsequently are more desirable for effective outcomes. Below we inspect various types of nucleic acids based on their ability to derive distinct types of functional outcomes.

### Transgene Expression

In the original gene therapy approach, a gene of interest was introduced into the cells to tap into the native machinery to produce the therapeutic protein, in order to replace a defective version (such as a mutated, non-functional protein) or supplement an additional capability such as morphogen-induced tissue regeneration. The use of viruses has been favored to ensure effective (increased uptake) and long-lasting (chromosomal integration) transgene expression, but using plasmid DNA (pDNA) and other naked nucleic acids eliminates several undesirable viral effects, as long as the delivery is effective. It has been possible to design tissue-specific, inducible, minimally-recognizable and “mini” pDNAs to overcome various limitations of the initial pDNA configurations. In addition to circular pDNA, it is possible to rely on other configurations of functional genes; the expression cassettes may come in various molecular weights, conformation and topologies (Sum et al., 2014). Lower molecular weight mini pDNA vectors, both linear and circular conformations, show better cytoplasmic diffusion compared to their parental plasmid precursors. Ministring DNA vectors, which are mini linear covalently closed DNA vectors, demonstrate improved cellular uptake, transfection efficiency, and target gene expression in comparison to isogenic minicircle DNA, which are mini circular covalently closed DNA vectors, of the same size and structure as the ministring DNA (Nafissi et al., 2014). Simultaneous delivery of two pDNAs is employed in the *sleeping beauty* (SB) transposon system, wherein one pDNA carries the SB transposase gene while the other pDNA carries the gene of interest flanked by the transposase recognizable terminal inverted repeats (TIRs). The capability of the transposon system to permanently insert transgene constructs in the host genome and relatively superior biosafety profile, makes the SB approach advantageous over non-integrating non-viral vectors and viruses, respectively (Kebriaei et al., 2017; Tipanee et al., 2017a). We (Hsu and Uludag, 2008) and others (Dhanoya et al., 2011) have previously shown that polymeric gene carriers can condense and deliver widely different DNA molecules. How cells process different DNA molecules is an understudied area with important implications in transgene expression efficiency; comparative assessment of uptake of nucleic acid complexes (Hsu and Uludag, 2008; Symens et al., 2013; Levacic et al., 2017), nuclear localization (Dhanoya et al., 2011), intracellular diffusion, and increased propensity for dissociation and/or endosomal release (Ribeiro et al., 2012) remains to be fully investigated especially in clinically relevant cells, but the ease of industrial expansion favors pDNA of various configurations for large scale applications. To overcome any transcriptional barriers (such as nuclear targeting and recognition by transcription factors), recent attempts have focused on delivering mRNA that can remain in cytoplasm and access the translational machinery readily (see section mRNA Delivery to Replace pDNA Therapy for more details on mRNA delivery).

### Gene Silencing

In order to silence unwanted or undesirable genes, antisense oligonucleotides (ASO; 16–20 nucleotide long single-stranded DNAs) that “neutralize” and block translation of target mRNAs were initially pursued that rely on *ex vivo* chemical synthesis and delivery. Apart from silencing defective genes, ASOs are finding applications in restoring the correct splicing patterns of pre-mRNAs that possess aberrant sequence elements involved in splicing or aberrant splice sites, as well as in altering expression levels of splice variants to affect a change in the function of a gene. This is executed by designing ASOs complementary to specific splice sites, thus blocking spliceosome assembly at the targeted splice site, which thereby leads to a shift of the splicing machinery to another splice site. The ASOs are expected to be capable of entering the cell nucleus, which is the site for pre-mRNA splicing. This is achieved by employing nucleotide bases, sugars and internucleotide linkages with modified chemistries (Kole and Sazani, 2001). A notable example of utilizing ASOs in this modality is the development of exon skipping therapy, wherein ASOs are used to restore the reading frame by skipping an exon or exons containing disease-driving mutations. Exon skipping therapy has been well-explored clinically in the context of Duchenne Muscular Dystrophy (Cirak et al., 2011; Goemans et al., 2016; Mendell et al., 2016; Aartsma-Rus et al., 2017), with an ASO drug receiving FDA approval in 2016 (Sarepta Therapeutics)^1^.

The endogenous RNA interference (RNAi) mechanism has been adopted for therapy by silencing genes based on blockage and/or degradation of corresponding mRNAs. RNAi can be implemented with synthetic short interfering RNAs (siRNAs; 19–27 nucleotide long double-stranded RNAs), as well as *in situ* production of silencer molecules (short hairpin RNAs; shRNAs) through typical pDNA-based expression vectors. While the latter relies on nuclear targeting for efficient expression, siRNAs can be delivered to the cytoplasmic space to engage the RNA-induced silencing complex (RISC) directly with minimal processing by host cells. To achieve sustained silencing of gene expression, siRNA needs to be continually supplied exogenously, or stable integration and expression is required in the case of shRNAs. The exciting possibilities with RNAi was recently (2018) confirmed with the FDA approval of the first siRNA based drug (Patisiran by Alnylam) to treat the nerve damage caused by the rare disease hereditary transthyretin-mediated amyloidosis (hATTR) in adults. To further regulate gene expression, endogenous miRs, the non-coding single stranded RNAs with 19 to 25 nucleotides and mis-matched base pairing, can be introduced into host cells either to augment or “mimic” a particular miR. In cases where the elevated miRs themselves are the cause of underlying pathophysiology and need to be down-regulated, single stranded RNA molecules with sequence complementary to a target miR, an anti-miR, could be deployed.

Gene suppression can also be executed by utilizing a subset of RNAs, called ribozymes or catalytic RNAs, that possess enzymatic action and can cleave target mRNA with high specificity to prevent protein translation (Abera et al., 2012). Besides utilizing artificially engineered ribozymes exogenously, significant efforts have been directed toward intracellular expression of these molecules. Expression cassettes with different kinds of promoters (e.g., long-acting, cell-specific, inducible, etc.) have been explored to optimize activity of the expressed ribozyme *in vivo*. To enhance stability and ensure proper folding into its active structure, ribozymes are expressed as part of a larger transcript, called carrier RNA, the sequence of which is carefully chosen so that the resulting transcript not only adopts a secondary structure that does not impede ribozyme activity but is also stable (Cagnon et al., 1995; Good et al., 1997; Prislei et al., 1997). Apart from downregulation of aberrant genes by *trans*-cleaving ribozymes, pathogenic genes can be repaired and/or reprogrammed by another subset called *trans*-splicing ribozymes. In the repair modality, mutated genes are cleaved and replaced by wild-type RNA sequences to yield properly functioning genes while maintaining the spatial and temporal gene expression patterns in cells and tissues (Byun et al., 2003; Shin et al., 2004). In malignancies and pathological tissue induction, where multiple pathways are dysregulated, repairing a single gene may not be sufficiently effective and hence require expression of multiple therapeutic genes. *Trans*-splicing ribozymes with reprogramming capabilities have been developed that not only remove viral transcripts and tumor-related genes but also induce cell death leading to elimination of virus-infected and cancer cells (Won and Lee, 2012; Carter et al., 2014; Kim et al., 2017). Since reprogramming genes comes with a great risk of unconstrained expression and unintended gene removal, it is paramount to introduce elements that maintain a check on the activity of *trans*-splicing ribozymes. This has been achieved by incorporating miR target sites or hypoxia-inducible elements in ribozymes to regulate their activity in a miR expression status-dependent manner or the ambient cellular environment, respectively. The potential of *trans*-splicing ribozymes in gene therapy and as anti-viral and anti-cancer tools has been reviewed elsewhere (Lee et al., 2018).

### “Sponging” Nucleic Acids

Long non-coding RNAs (lncRNA) have been recently identified whose sole function seem to sequester and alleviate the effects of intracellular molecules responsible for undesirable changes. It may be possible to alter the activity of specific DNA, RNA, and protein targets by deploying lncRNAs ‘sponges.' Sponging may be additionally undertaken by so called circular RNAs (circRNAs) distinct from linear lncRNAs and miRs, featuring higher cellular stability due to the absence of free ends and resistance to exonucleolytic degradation. CircRNAs may harbor one or more binding sites for a single miR or possess binding sites for multiple miRs, thereby regulating entire miR families (Panda et al., 2016; Chen et al., 2017; Hsiao et al., 2017). Besides miR sponges, circRNAs harboring a high density of binding sites for one or more RNA-binding proteins, serve as protein sponges, thereby modulating levels of target proteins which leads to changes in downstream intracellular events (Ashwal-Fluss et al., 2014; Abdelmohsen et al., 2017). Alternatively, they may operate as protein scaffolds facilitating contact between two or more proteins when they possess binding sites for enzymes and their substrates (Du et al., 2017). Their sponging capacity can be harnessed by engineering them to include combinations of miR and protein binding sites to target specific disease profiles. Since they can also serve as templates for protein expression in the presence of appropriate translation signals (Legnini et al., 2017; Pamudurti et al., 2017; Yang et al., 2017), expression cassettes for therapeutic proteins can be incorporated into circRNAs for gene therapy. Research on the practical implementation of therapeutic circRNAs is still in its infancy, with significant room for exploratory studies to realize their therapeutic potential.

Other nucleic acids that can affect cellular events by sequestering biomolecules are DNA and RNA aptamers. They are 56–120 nucleotides long, single-stranded synthetic oligonucleotides that can bind to various targets including small organic compounds and proteins, both intracellular and extracellular, with high affinity and specificity. They can fold into three-dimensional (3D) structures for binding to their target proteins through structural recognition and inhibit their interactions with other molecules in a manner similar to protein antagonists and antibodies, thereby serving as decoys. Their ability to recognize and bind highly structured nucleic acid targets lends them a unique functionality that may be more advantageous over previously mentioned agents (i.e., ASOs, siRNAs, miRs, ribozymes etc.). While extracellular molecules can be targeted by exogenous aptamers with relative ease, *in situ* production of aptamers through expression vectors has been explored for more efficient targeting of intracellular molecules (aptly known as “intramers”) (Chaloin et al., 2002; Choi et al., 2006; Mi et al., 2006). Many different promoter systems and expression cassettes have been designed to obtain high intracellular levels and sustained expression of aptamers. The functionality of endogenously expressed RNA aptamers can be impaired by flanking sequences in the RNA aptamer transcript, as interaction with them can hamper proper folding and render them inert (Sullenger et al., 1990; Blind et al., 1999; Martell et al., 2002). To overcome this limitation, sequences coding for ribozymes have been incorporated into the vector, which upon expression cleaves the aptamer from the nascent RNA transcript (Joshi and Prasad, 2002; Nishikawa et al., 2003). A strategy to direct intramers to extra-nuclear compartments and localize them close to their target(s) is to include nuclear export signal sequences, which enable translocation of expressed aptamers through nuclear pore complex (Grimm et al., 1997; Hamm and Fornerod, 2000). Control over intramer activity can be attained by employing a bi-aptamer construct where one aptamer serves as a sensor for the biological trigger and the other aptamer exerts inhibitory action. The expression cassette for these trigger-inducible systems include a connection sequence between the two aptamer sequences, resulting in a functional fusion product (Ausländer et al., 2011). In a separate avenue of exploration, the capacity of aptamers to bind a diverse range of targets has been extensively exploited for derivatization of NP delivery systems for site- or target-specific delivery. The success of this strategy is evident by numerous therapeutic RNA aptamers undergoing clinical trials (Sundaram et al., 2013; Sridharan and Gogtay, 2016).

### Gene Editing

Recent advances in gene editing technology based on clustered regularly interspaced short palindromic repeat (CRISPR)/Cas9 nuclease is providing exciting possibilities but also raising the bar for biomaterial-mediated delivery. The CRISPR/Cas9 system requires a single guide RNA (gRNA) and the Cas9 nuclease to undertake gene editing. For practical implementation, alongside the gRNA, the Cas9 nuclease may be delivered directly as a protein, as a pDNA cassette for protein expression in host cell, or as a “translatable” mRNA (Lino et al., 2018; Wang et al., 2018). Co-delivery of different cargoes presents a great challenge in designing an optimal delivery vehicle due to differences in physical structure and chemical properties of the different types of cargo. For instance, in contrast to anionic nucleic acids, the Cas9 protein is cationic (Sun et al., 2015), so that biomaterials have to accommodate the contrasting features of the cargo during the packaging and delivery. Biomaterials capable of optimally complexing long-string like transposase mRNA will be different from carriers that optimally interact with gRNA, so that mutually compatible carriers are likely to require concerted efforts. The situation is analogous to the SB transposon system, where supplying transposase from *in situ* translated mRNA (preferable to avoid the risk of chromosomal integration) (Wiehe et al., 2007; Holstein et al., 2018) instead of pDNA expression cassette will require distinct optimization of the biomaterial carrier to accomodate both types of cargo. We had previously articulated on the importance of delivering multiple agents as being the preferred approach in the case of most pathophysiologies (e.g., cancers where the internal physiology is altered in several respects) (KC et al., 2017). Deploying a single agent, while convenient for pharmacological development, may not be as effective in controlling the disease in these cases. Augmenting a defective gene may need to be undertaken while suppressing other mediators or augmenting other genes and miRs, for which combinatorial delivery of different nucleic acids will be needed (KC et al., 2017). In attempts to co-deliver a pDNA and siRNA, for example, one is faced with the delivery of a long flexible DNA molecule (>3000 bp) and a short rigid RNA molecule (<30 bp). Composite materials or chemically-distinct delivery vehicles capable of self-assembling into functional structures with different nucleic acids are needed to this end. While technically challenging, undertaking combinatorial delivery may offer the advantage of enhancing the biosafety and toxicity of certain vectors given the improvements in efficacy and the need to deploy a lower dose of the therapeutic agents.

### “Hybrid” Nucleic Acids for Responsive Systems

The simplicity of the four-nucleotide chemistry and Watson-Crick base pairing provides significant room for flexibility, as a consequence of which generation of different combinations of polynucleotides has been feasible. Chimeric constructs developed by combining different types of nucleic acids allows us to benefit from their respective desirable characteristics and diverse functionalities. The targeting capability of aptamers has been harnessed by conjugating them with ASOs, siRNAs, shRNAs, and miRs for cell-specific delivery and subsequent gene silencing (Soldevilla et al., 2018). Additionally, the unique binding capability of aptamers to small molecules has been employed to generate RNA-based regulators that integrate sensing functions and endogenous gene regulation, through ligand-responsive aptamer-miR chimeras (Beisel et al., 2011). Molecular switches that turn on/off a certain function contingent upon a physiological signal has been manifested by integrating aptamers with ribozymes, known as aptazymes. It has been shown that target interaction with aptamer induces adaptive folding around the bound target leading to adoption of a distinct conformation as well as further stabilization of adjacent helical domains. This stabilization affects the conformation of the attached ribozyme leading to a switch in its activity (Famulok et al., 2007). These triggerable systems, once optimized, permit better control over relatively complicated therapeutic strategies that target regulatory networks or genome reprogramming, as reported in a study where an aptazyme was embedded within the gRNA of a CRISPR/Cas9 system (Tang et al., 2017). By inserting multiple aptamer sequences harboring specificities for different ligands, it may be possible to obtain more precise control over genome editing and subsequently over spatial and temporal gene expression patterns to rectify diseased states.

Apart from aptamers, DNA could also serve as a delivery vehicle when folded into nanostructures through the scaffolding DNA origami technique, as demonstrated by successful *in vivo* delivery of siRNA by a DNA tetrahedron (Lee et al., 2012). Although the added functionality imparted by hybridizing different nucleic acids has a great appeal, especially in the context of gene therapy, it is critical to ensure that integration does not hamper the biological activity of either molecule. Other practical considerations such as synthesis cost:yield ratio, benefit:cost ratio, suitability and need for the intended application should also be evaluated during design and creation of these types of hybrid constructs.

### Nucleic Acids Without Carriers

Finally, we note that numerous clinical studies are underway where new generation of nucleic acids are being deployed without the use of a carrier (Table 1). Presumably, the rationale behind this approach is to avoid the introduction of synthetic carrier materials which may not be degraded at times and hence may accumulate leading to adverse effects. Carrier-free delivery eliminates the process of development and optimization of a delivery vehicle, but however poses its own set of challenges. Several factors such as poor permeability to cell membranes due to their anionic nature, rapid clearance owing to their small size, and susceptibility to degradation by ubiquitous nucleases, render the nucleic acids unfavorable in their native form. To overcome these physiological barriers, chemical modifications have been incorporated in their sugar-phosphate backbones as well as sugar and base moieties. While these modifications confer desirable attributes such as enhanced stability, nuclease resistance, target binding affinity, and reduced immune stimulation, it is crucial to include them in a balanced proportion to circumvent loss of potency. Besides chemical modifications, nucleic acids are conjugated with lipid moieties or polyethylene glycol (PEG) or small cationic proteins to aid in increasing their size, traversing the complex physiological milieu, enhancing circulation half-life, and eventually potentiating their therapeutic efficacy. Practically, utilization of naked nucleic acids seems most appropriate for localized treatment strategies, as is evident from Table 1, where ~60% of the indicated clinical studies employ subcutaneous, intramuscular, intravitreal or other localized routes of administration. In these modes, they are challenged with relatively less physiological hurdles to reach their site of action and carry out their activity. Accordingly, carrier-free nucleic acid therapeutics are suitable for external and/or easily accessible tissues such as ocular, epidermal, pancreatic, pulmonary, and colonic tissues. The route of administration has a significant influence on drug biodistribution, bioavailability, and eventually its therapeutic efficacy, so that initial focus on the development of nucleic acid therapeutics for ailments of the eye, skin, and muscle are understandable. Local administration also allows for implementing gene medicines by patients through eye drops and nasal sprays. However, for more deep-seated maladies, naked nucleic acids may not be satisfactory as in this case they need to be administered systemically and are required to seek out the diseased tissue in the complex *in vivo* environment to be effective. For this, they need to be equipped with the right elements to identify target tissues and evade degradation, while still be biologically active. Incorporating chemical modifications can adversely effect potency, making it incumbent to utilize carriers for nucleic acid therapeutics intended for these applications. While viruses are efficient and effective carriers, significant effort has been invested in developing safer, less immunogenic non-viral techniques and biomaterials for delivering nucleic acid therapeutics in hematological malignancies, as reviewed elsewhere (Ansari et al., 2017).

**Table 1:** Carrier-free nucleic acid therapeutics in clinical trials.

**Drug**	**Nucleic acid**	**Target**	**Route of administration**	**Indication**
SYL1001	siRNA	TRPV1	Ophthalmic drops	Dry eye syndrome
ALN-GO1 (Lumasiran)	GalNAc-siRNA	HAO1	Subcutaneous	Primary hyperoxaluria type I
Bevasiranib	siRNA	VEGF	Intravitreal injection	AMD/DME
SYL040012	siRNA	ADRB2	Ophthalmic drops	Intraocular pressure
PF-655	siRNA	RTP801	Intravitreal injection	AMD/DME
I5NP (QPI-1002)	siRNA	P53	Intravenous	AKI and DGF
DCR-HBVS	GalNAc-siRNA	HBV	Subcutaneous	Chronic hepatitis B
DCR-PHXC	GalNAc-siRNA	LDHA	Subcutaneous	Primary hyperoxaluria
BMT101	Lipophilic compound-siRNA	CTGF	Intradermal injection	Hypertrophic scars
QPI-1007	siRNA	Caspase 2	Intravitreal injection	NAION
AGN-745	siRNA	VEGFR-1	Intravitreal injection	AMD
TD101	siRNA	KRT6A	Intralesional injection	Pachyonychia congenita
ALN-RSV01	siRNA	RSV nucleocapsid	Nebulization or intranasal	RSV infection
SRP-4053	ASO	Exon 53 skipping in dystrophin gene	Intravenous	DMD
GTI-2040	ASO	RNR	Intravenous	Leukemia, MDS, solid tumors
NS-065/NCNP-01	ASO	Exon 53 skipping in dystrophin gene	Intravenous	DMD
EZN-2968	ASO	HIF-1α	Intravenous	Advanced solid tumors and lymphoma
TPI ASM8	Two ASOs	CCR3 and β chain of IL3, IL5, and GM-CSF receptors	Inhalation	Asthma
ISIS 104838	ASO	TNF-α	Subcutaneous	Rheumatoid arthritis
OGX-427 (Apatorsen)	ASO	Hsp27	Intravenous	Prostate, ovarian, breast, bladder cancer, and SCLC
G3139 (Oblimersen)	ASO	Bcl-2	Intravenous or subcutaneous	Solid tumors, multiple myeloma, DLBCL and CLL
AZD4785	ASO	KRAS	Intravenous	Advanced solid tumors
AZD5312	ASO	Androgen receptor	Intravenous	Advanced solid tumors
ISIS 5132	ASO	c-Raf kinase	Intravenous	Metastatic breast cancer
ISIS 3521	ASO	PKC α	Intravenous	Metastatic breast cancer
AZD9150	ASO	STAT3	Intravenous	Gastrointestinal, ovarian cancer, hepatocellular carcinoma, and DLBCL
ISIS 183750	ASO	eIF4E	Intravenous	Colorectal cancer
DS-5141b	ASO	Exon 45 skipping in dystrophin gene	Subcutaneous	DMD
AVI-4658	ASO	Exon 51 skipping in dystrophin gene	Intramuscular	DMD
EZN-4176	ASO	Androgen receptor	Intravenous	Prostate cancer
ISTH0036	ASO	TGF-β2	Intravitreal injection	Glaucoma
AEG35156	ASO	XIAP	Intravenous	Pancreatic and breast cancer
RG6042	ASO	Huntingtin	Intrathecal injection	Huntington's disease
WVE-120102	ASO	Huntingtin	Intrathecal injection	Huntington's disease
WVE-210201	ASO	Exon 51 skipping in dystrophin gene	Intravenous	DMD
OGX-011 (Custirsen)	ASO	Clusterin	Intravenous	Solid tumors
RO7070179	ASO	HIF-1α	Intravenous	Hepatocellular carcinoma
ISIS 396443 (Nusinersen)	ASO	SMN2	Intrathecal injection	Spinal muscular atrophy
Kynamro^*^ (Mipomersen)	ASO	ApoB	Subcutaneous	Homozygous familial hypercholesterolemia
ISIS 420915	ASO	Transthyretin	Subcutaneous	Cardiac amyloidosis
ISIS 113715	ASO	PTP-1B	Subcutaneous	Type 2 diabetes mellitus
ISIS 2302	ASO	ICAM-1	Intravenous	Crohn's disease
Cenersen	ASO	P53	Intravenous	MDS
IONIS-STAT3Rx	ASO	STAT3	Intravenous	DLBCL and advanced lymphoma
IONIS-ENaCRx	ASO	ENaC	Inhalation	Healthy volunteers
IONIS FXI-LRx	ASO	Factor XI	Subcutaneous	Healthy volunteers
IONIS PKK-LRx	ASO	PKK	Subcutaneous	Healthy volunteers
IONIS APOC-III-LRx	GalNAc3-ASO	ApoC-III	Subcutaneous	Elevated triglycerides
SB101	DNAzyme	GATA-3 transcription factor	Inhalation	Asthma
SB012	DNAzyme	GATA-3 transcription factor	Rectal route	Ulcerative colitis
MRG-201	miR mimic	miR-29b	Intradermal	Pathologic fibrosis, keloids
SPC3649	AntimiR	miR-122	Subcutaneous	Hepatitis C
CV9104	50% free mRNA + 50% protamine/mRNA (2:1 w/w)	PSA, PSMA, PSCA, STEAP1, PAP, MUC1	Intradermal	Prostate cancer
CV9201	50% free mRNA + 50% protamine/mRNA (2:1 w/w)	NY-ESO1, MAGE-C1, MAGE-C2, survivin, 5T4	Intradermal	NSCLC
CV7201	Free and protamine/mRNA	Rabies virus glycoprotein	Intradermal or intramuscular	Rabies vaccine
iHIVARNA-01	mRNA	CD40L, CD70, caTLR4, HIV immunogen	Intranodal injection	HIV-1 infection
Tumor mRNA vaccine	mRNA	Melan A, MAGE A1, MAGE A3, survivin, gp100, tyrosinase	Intradermal or subcutaneous	Malignant melanoma
QR-421a	RNA-based oligonucleotide	Exon 13 skipping in USH2A gene	Intravitreal injection	Retinitis Pigmentosa
QR-110	RNA-based ASO	CEP290	Intravitreal injection	Leber's Congenital Amaurosis
QR-010	RNA-based ASO	CFTR	Intranasal	Cystic fibrosis
REG1	RNA aptamer and a PEG-RNA aptamer	Factor IXa	Intravenous	Acute coronary syndrome, coronary artery disease, PCI
AS1411	PEG-DNA aptamer	Nucleolin	Intravenous	AML and solid tumors
ARC1799	PEG-DNA aptamer	Von Willebrand factor	Intravenous	Von Willebrand disease, purpura, thrombotic thrombocytopenia, PCI, AMI, and thrombosis
NOX-E36	PEG-RNA aptamer	CCL2	Intravenous or subcutaneous	Chronic inflammatory diseases, type 2 diabetes mellitus, and SLE
NOX-A12	PEG-RNA aptamer	CXCL12	Intravenous	Stem cell transplantation, multiple myeloma, CLL, NHL, colorectal and pancreatic cancer
E10030	PEG-DNA aptamer	PDGF	Intravitreal injection	AMD and Von Hippel-Lindau Syndrome
ARC1905	PEG-RNA aptamer	Complement 5	Intravitreal injection	AMD and idiopathic polypoidal choroidal vasculopathy
NU172	DNA aptamer	Thrombin	Intravenous	Thrombosis
Macugen^*^ (Pegaptanib)	PEG-RNA aptamer	VEGF	Intravitreal injection	AMD/DME
ARC19499	PEG-RNA aptamer	TFP1	Intravenous or subcutaneous	Hemophilia
NOX-H94	PEG-RNA aptamer	Hepcidin peptide hormone	Intravenous	Anemia of chronic disease and end stage renal disease
Angiozyme	Ribozyme	VEGFR-1	Subcutaneous	Renal cancer
Heptazyme	Ribozyme	HCV IRES	Subcutaneous	Hepatitis C

The data is compiled from www.ClinicalTrials.gov based on nucleic acid keyword search and choosing the trials where no obvious carrier was used. FDA approved drugs are indicated with an^*^.

*TRPV1, Capsaicin receptor; GalNAc, N-acetylgalactosamine; HAO1, Hydroxyacid oxidase; VEGF, Vascular endothelial growth factor; AMD, Age related macular degeneration; DME, Diabetic macular edema; ADRB2, Beta-2 adrenergic receptor; RTP801, Pro-angiogenic factor; AKI, Acute kidney injury; DGF, Delayed graft function; HBV, Hepatitis B virus; LDHA, Lactate dehydrogenase A; CTGF, Connective tissue growth factor; NAION, Non-arteritic anterior ischemic optic neuropathy; VEGFR-1, Vascular endothelial growth factor receptor-1; KRT6A, Keratin 6a; RSV, Respiratory syncytial virus; DMD, Duchenne muscular dystrophy; RNR, Ribonucleotide reductase; MDS, Myelodysplastic syndrome; CCR3, Eotaxin receptor; IL, Interleukin; GM-CSF, Granulocyte-macrophage colony-stimulating factor; TNF-α, Tumor necrosis factor- α Hsp27, Heat shock protein 27; SCLC, Squamous cell lung cancer; Bcl-2, B-cell lymphoma 2; DLBCL, Diffuse large B-cell lymphoma; CLL, Chronic lymphocytic leukemia; CML, Chronic myeloid leukemia; KRAS - Ki-ras2 Kirsten rat sarcoma viral oncogene homolog; PKC α, Protein kinase C α; TGF-β2, Transforming growth factor-β2; HIF-1α, Hypoxia inducible factor-1α; XIAP, X-linked inhibitor of apoptosis; SMN2, Survival motor neuron 2; ApoB, Apolipoprotein B; PTP-1B, Protein tyrosine phosphatase 1B; ICAM-1, Intercellular adhesion molecule 1; STAT3, Signal transducer and activator of transcription 3; ENaC, Epithelial sodium channel; PKK, Protein kinase C-associated kinase; ApoC-III, Apolipoprotein C3; eIF4E, Eukaryotic translation initiation factor 4E; GATA-3, GATA binding protein 3; PSA, Prostate-specific antigen; PSMA, Prostate-specific membrane antigen; PSCA, Prostate stem cell antigen; STEAP1, Six transmembrane epithelial antigen of the prostate 1; PAP, Prostatic acid phosphatase; MUC1, Mucin 1; NY-ESO1, New York esophageal squamous cell carcinoma; MAGE, Melanoma antigen family; 5T4, Trophoblast glycoprotein; NSCLC, Non-small cell lung cancer; CD, Cluster of differentiation; TLR, Toll-like receptor; HIV, Human immunodeficiency virus; Gp100, Glycoprotein 100; USH2A, Usher syndrome type IIa; CEP290, Centrosomal protein 290; CFTR, Cystic fibrosis transmembrane conductance regulator; PCI, Percutaneous coronary intervention; AMI, Acute myocardial infarction; AML, Acute myeloid leukemia; CCL2, Chemokine (C-C motif) ligand 2; SLE, Systemic lupus erythematosus; CXCL12, C-X-C motif chemokine 12; NHL, Non-Hodgkin's lymphoma; PDGF, Platelet-derived growth factor; TFP1, Tissue factor pathway inhibitor; HCV IRES, Hepatitis C virus internal ribosome entry site*.

## Technology for Cellular Engineering: T-Cell Therapy as a Case in Point

The exciting developments in T-cell therapies are providing opportunities for biomaterials to implement a new type of gene medicine. T-lymphocytes are essential for adaptive immunity as they acquire T-cell receptors (TCRs) in the thymus to recognize foreign antigens from infectious pathogens as well as tumor antigens (Mitchison, 1955; Jorgensen et al., 1992; Park and Renier, 2010). Since 1980s, *ex-vivo* expanded T-cells have been used for treatment of diseases such as melanoma, cytomegalovirus and HIV (Rosenberg et al., 1988; Riddell et al., 1992; Levine et al., 2002). The initial deployment of T-cells required sorting and expansion of allogeneic or autologous lymphocytes for their reintroduction into patients, yet generation of disease-specific T-cells is cumbersome as patients usually express limited numbers of cells that are reactive against the specific target (Sadelain et al., 2003; Park and Renier, 2010). Using allogeneic T-cells and in some cases autologous T-cells led to high risks of developing graft-vs.-host disease and rejection of infused T-cells. Relying on naturally expressed TCRs requires tumor antigens to be presented by specific major histocompatibility complexes (MHC), which are usually down-regulated or dysfunctional in many tumors besides being very specific to each patient (Hicklin et al., 1999; Khong and Restifo, 2002; Park and Renier, 2010).

Engineered T-cells have emerged to better control the safety and effectiveness of T-cell therapies particularly controlling antigen targeting and T-cell function (Sadelain et al., 2003). They represent one of the most advanced therapeutic options as they are a “living drug” which combine major advances in antibody engineering, vaccination and transplantation (Lim and June, 2017). Two T-cell based therapies recently approved by the FDA (National Cancer Institute, 2017), axicabtagene ciloleucel (Yescarta™)^2^ and tisagenlecleucel (Kymriah™)^3^, are genetically modified cells to express Chimeric Antigen Receptors (CARs) against CD19, an antigen present throughout the B-cell lineage and one of the first targets for development of monoclonal antibodies (mAb) for B-cell malignancies (Engel et al., 1995; Katz and Herishanu, 2014; Park et al., 2016). Along with targeting “liquid” cancers, they are now being explored to target ‘solid' cancers, as well as infectious diseases or undesired immune responses with >250 ongoing clinical trials (Scholler et al., 2012; American Association for Cancer Research, 2017; Maldini et al., 2018).

T-cells have been primarily modified to express CARs by viral gene transfer; in cases of Yescarta^TM^ and Kymriah™, replication-defective gammaretrovirus and lentivirus vectors, respectively, were used for gene transfer (Hu and Pathak, 2000; Sadelain, 2017; Zhang et al., 2017), which enabled permanent transgene insertion into the genome (Hu and Pathak, 2000). However, retroviral gene transfer has been associated with high risk of insertional mutagenesis in the past, especially when vectors get inserted close to growth-control genes, leading to oncogenesis, immune reactions, and other toxicities (Hacein-Bey-Abina et al., 2008; Wang et al., 2008, 2015). The production of viral vectors is also laborious, with production times ranging from 2 weeks to 6 months and differences in batches or sources making it difficult to compare and replicate (Przybylowski et al., 2005; Ivics et al., 2009; Levine et al., 2016; Kebriaei et al., 2017; Zhang et al., 2017). As a result of these drawbacks, non-viral vectors that are easier to synthesize, cheaper, less toxic and more consistent to produce are being constantly developed to match the effectiveness of viral vectors (Table 2). SB transposons, just as retroviral vectors, can integrate themselves in the genome and address the issue of longevity of expression (Ivics et al., 2009). The SB transposon system was the first one to be effective in vertebrate cells and since then other transposons that are more active in some cell lines, such as the piggyback transposon, have been investigated (Wu et al., 2006; Muñoz-López and García-Pérez, 2010). Transposons rely on TIRs that are recognized by transposases to “cut” and “paste” the gene at desired destinations. Thus, a transposon vector with the gene of interest with the TIRs and a transposase-coding pDNA or mRNA need to be delivered to target cells (Yant et al., 2000; Wu et al., 2006). While transposon systems could be simpler and more predictable with lower risk of immunogenicity (Walisko et al., 2008), transposable elements are not free of risks of genotoxicity and they still rely on carriers for transport through the cell membrane. Viral vectors are still being used for transposons with the same challenges discussed before (Doudna and Charpentier, 2014; Boehme et al., 2016; Richter et al., 2016). Other gene editing technologies include designer nucleases, including zinc finger nucleases (ZFN), transcription activator-like effector nucleases (TALEN) and CRISPR/Cas9 system which induce double strand breaks in a target site followed by the addition of a gene of interest (Urnov et al., 2010; Gaj et al., 2013; Jung and Lee, 2018). The CRISPR/Cas9 system is already being tested in clinical trials in China and CAR T-cell engineering in the US (Svoboda et al., 2018). The designer nucleases depend on cellular enzymes for gene insertion that require dividing cells in contrast to some integrating viruses and transposon systems that can also target non-dividing cells (Di Stasi et al., 2011).

**Table 2:** Broad comparison of viral and non-viral transfection vectors.

**Criteria**	**Viral**	**Non-viral**
Immunogenicity, Inflammation	High-medium risk	Medium-low risk
Mutagenesis	High-medium risk	Medium-low risk
Vector production	Laborious, batch to batch variability	Ranges in difficulty
Transfection efficiency	High efficiency	Medium-low efficiency
Duration of gene expression	Long term	Medium, transient

One of the main challenges associated with shifting to non-viral methods for transfection of T-cells is the inability of non-viral systems to match the efficiency of viral systems, especially in terms of longevity of gene expression. However, the transient expression by non-viral vectors may be advantageous as it may lead to reduced side effects and complications for patients (Hardee et al., 2017). Another challenge for non-viral vectors in T-cell engineering is less than optimal *in vivo* targeting and continuous stimulation that must be provided by the material to the cells, which is inherently difficult to achieve as these cells are present in suspension, are constantly dividing and usually exist in an immunosuppressive environment (Zheng et al., 2013; Ansari et al., 2017). Thus, innovative biomaterials that find solutions to these two challenges are of paramount importance in the field and can also serve as a point of reference for biomaterials targeting various cells besides T-cells. The alternative to viral modification of T-cells is commonly based on membrane pore-inducing electroporation/nucleofection without any carriers. Ramanayake et al. recently compared the average costs of viral delivery ($3-500,000) with an electroporation/transposon approach ($6,000) to produce CAR T-cells under GMP conditions. By optimizing electroporation conditions, modified CAR T-cells persisted in the peripheral blood for >3 weeks and transgene expression was >50% (Wells, 2004). A safety guard included in their transposon sequence was the inducible caspase 9 suicide gene, which directs targeted elimination of engineered T-cells by administration of a small molecule (Wang et al., 2017). Some drawbacks of electroporation, however, are toxicity and difficulty for *in vivo* applications due to limited access to target sites (Holstein et al., 2018). Longer *ex vivo* expansion might be required to allow cells to recover from undesirable consequences of electroporation, since grafting nucleofected hematopoietic cells in a preclinical model was improved with longer culture times (Holstein et al., 2018). An alternative approach to electroporation is “cell squeezing” using microfluidic devices that rely on rapid mechanical deformation of cells to passively introduce genes and materials of interest (Sharei et al., 2013a,b).

In a similar manner, as more precise control is desired for immunomodulation of T-cells, diverse biomaterials and nanotechnologies have emerged as platforms to address major stages and challenges of CAR T-cell development, namely *ex vivo* and *in vivo* expansion of cells and CAR gene delivery (Table 2). The majority of T-cell therapies involve *ex vivo* expansion of modified/to be modified cells but not all expanded T-cells have the same therapeutic efficacy (Fraietta et al., 2018). T-cell expansion most commonly employs commercially available polystyrene microbeads (Dynabeads) that aim to simulate the action of antigen presenting cells (APCs) targeting T-cell activation through CD3 and CD28 stimulus and IL-2 supplementation (Kalamasz et al., 2004; Hollyman et al., 2009; Li and Kurlander, 2010). However, these non-degradable beads need to be separated prior to cell delivery to patients and they also dysregulate some T-cell functions as their mode of action is not as close as APC activation. Other alternatives to naturally derived APCs include poly(lactide-co-glycolide) (PLGA) microparticles (Steenblock and Fahmy, 2008), phosphatidylcholine and cholesterol liposomal systems (Prakken et al., 2000), paramagnetic iron-dextran NPs (Perica et al., 2014), polydimethylsiloxane (PDMS) microbeads (Lambert et al., 2017) and carbon nanotubes composites with PLGA NPs (Fadel et al., 2014), highlighting the compatibility of various systems with T-cell stimulation and also how different physiochemical properties of the particles may have different efficiencies on T-cell expansion. Besides the use of NP systems, 3D scaffolds have also been developed which can be used as implants at tumor sites. These systems have included commercial Matrigel and polystyrene scaffolds (Pérez Del Río et al., 2018), mesoporous silica microrods with supported lipid bilayer composites (Cheung et al., 2018), 3D-printed polycaprolactone lattices (Delalat et al., 2017), alginate scaffolds (Stephan et al., 2014) and injectable polyisocyanopeptide and PEGylated chitosan hydrogels (Tsao et al., 2014; Weiden et al., 2018a). With such broad possibilities, the versatility of biomaterials to design biomimetic systems to effectively expand T-cells is evident. Scaffolds have also been used as *in vivo* immunomodulation niches promoting sustained release and expansion of T-cells directly at tumor sites. For example, an alginate scaffold was reported that delivered T-cells and a STING (Stimulator of Interferon Genes) agonist, serving as a vaccine in pancreatic and melanoma mice models (Smith et al., 2017a). Co-delivery of T-cells and STING agonist not only eradicated the tumors in some mice, but also enabled the cured mice to develop a systemic antitumor immune response and resistance to metastasis when re-challenged with pancreatic tumor cells (Kim et al., 2014). One of the limitations of implantable scaffolds is that they require surgery to be introduced to desired sites, but to address this, injectable formulations that form scaffolds *in situ* are being developed. Injectable mesoporous silica rods were able to spontaneously form scaffolds, recruit APCs and subsequently elicit specific T-cell responses (Kim et al., 2014).

To deliver the CAR genes, synthetic carriers have also been developed (Table 3) with the aim of reducing viral-induced reactions, while increasing delivery loads and ease of manufacture (Zhou et al., 2017). Synthetic carriers may need to be modified with targeting antibodies, peptides or recombinant molecules that augment their transfection specificity in hard-to-transfect cells growing in suspension (Liu et al., 2018). So far, lipid (Moon et al., 2011) and polymeric (Smith et al., 2017b; Olden et al., 2018) delivery systems have been used for generating CAR T-cells with targeting capacity inducing tumor regression in a mouse model (Smith et al., 2017b). To our knowledge only two groups have reported *in vivo* generation of CAR T-cells, the Buchholz group at the Paul-Ehrlich-Institut and the Stephan group at the Fred Hutchinson Cancer Research Center. The Buchholz group reported a lentiviral approach targeting CD8 receptors and the Stephan group utilized poly(β-amino ester) NPs, both successfully generating CD19-CAR T cells *in vivo* (Smith et al., 2017b; Pfeiffer et al., 2018). Comparing the two approaches, the lentiviral approach allowed for greater percentage of CAR T-cell generation of up to 35% in blood and the synthetic NPs reported up to ~20% transfection. In addition, both studies compared their *in vivo* targeting systems to infusions with conventionally generated CAR T-cells *ex-vivo* and did not find significant differences between the two treatments. This opens up a new avenue to increase the efficiency of CAR T-cell engineering and avoid the cumbersome *ex vivo* expansion and reprogramming steps. It is to be noted, however, that even though both approaches established significant advances in the efficiency of CAR T-cell generation, they still encountered some of the main challenges of the therapy including B-cell depletion and signs of cytokine release syndrome with the use of lentivirus. B-cell depletion arises since CD-19 is not only present in leukemic cells but also in non-leukemic B-cells, highlighting the hurdle of finding the right antigen to target.

**Table 3:** Emergent biomaterial approaches for T-cell therapies.

**Material**	**Approach**	**References**
***ANTIGEN PRESENTING*** **PARTICLES FOR EXPANSION OF T-CELLS**		
Superparamagnetic beads (Dynabeads)	Conjugated to anti-CD3 and anti-CD28 to stimulate antigen specific T-cell expansion *ex-vivo*	Kalamasz et al., 2004
PLGA microparticles	Sustained IL-2 release to stimulate CD8+ T-cell expansion	Steenblock and Fahmy, 2008
Phosphatidylcholine liposome	Incorporated MHC II and highlighted how a synthetic system could mimic APC and T-cell interactions	Prakken et al., 2000
Iron dextran NPs	Utilized an external magnetic field to drive particle aggregation and enhance T-cell activation	Perica et al., 2014
PDMS microbeads	Soft elastomer formulation conjugated to anti-CD3 and anti-CD28	Lambert et al., 2017
Carbon nanotubes and PLGA composite	Composite system to cluster antigen presentation and release IL-2	Fadel et al., 2014
**SCAFFOLDS FOR EXPANSION OF T-CELLS**		
3D Polystyrene and Matrigel scaffolds	3D culture with polystyrene or Matrigel sustained superior proliferation of T-cells than suspension systems	Pérez Del Río et al., 2018
Fluid lipid bilayer on mesoporous silica rods	Combined fluidity of lipids on a solid platform that could present surface and soluble stimulus to T-cells	Cheung et al., 2018
3D printed polycaprolactone lattices	Printed scaffold with high reproducibility and scalability; superior than nanoparticle T-cell expansion	Delalat et al., 2017
Alginate scaffold modified with collagen-mimetic peptide	Introduced T-cells into mice tumor models using the alginate scaffold and prevented tumor relapse	Stephan et al., 2014
Polyisocyanopeptide hydrogel	Injectable thermo-responsive scaffolds that allowed *in-vivo* T-cell survival and migration	Weiden et al., 2018a
PEG-g-Chitosan Hydrogel (PCgel)	PCgel was compared to Matrigel and allowed for enhanced migration of T-cells targeting glioblastoma	Tsao et al., 2014
Alginate scaffold with collagen-mimetic peptide and adjuvant silica microparticles	Combined the release of T-cells with adjuvant compounds to elicit a local and systemic response	Smith et al., 2017a
Mesoporous silica rod assembled scaffold	Macroporous scaffold formed *in situ* recruited and modulated immune cells *in vivo*	Kim et al., 2014
**BIOMATERIALS FOR GENETIC MODIFICATION OF T-CELLS**		
Cationic pHEMA-g-pDMAEMA polymer	Highlighted different architectures of polymeric delivery systems achieving maximum transfection with comb and sunflower shaped polymers in primary T cells	Olden et al., 2018
Poly(B-amino) ester polymer	First time CAR T-cells developed *in vivo* by a nanoparticle system. Targeting ligands allowed for comparable survival improvement to conventional T-cell adoptive transfer.	Smith et al., 2017b

As more advances are reported for CAR T-cell technologies, some common challenges have emerged. The treatment of solid tumors is one such major challenge, as the tumor microenvironment is highly immunosuppressive due to combination of down-regulated tumor antigens and T-cell suppression (Joyce and Fearon, 2015; Cheung et al., 2018). However, the *in situ* scaffold approaches aim to reverse the immunosuppressive environment by sustained release of cytokines for recruitment of immune cells at tumor sites. Once the cells are transferred to patients, T-cells act autonomously and so far it is very difficult to control their actions and unwanted side effects *in-situ* [e.g., cytokine release syndrome, neurotoxicity or B-cell aplasia (Yant et al., 2000)], so that feedback systems are needed to better control the therapy (Lim and June, 2017). As CAR T-cell technologies continue to progress, an inter-disciplinary effort must be made to address some of the pressing challenges that include their mode of delivery and expansion, migration, and mechanism of action so as to exert their action on tumor cells while sparing the normal cells.

## Biomaterials and Emerging Nucleic Acid Technologies

Biomaterials have been an integral part of emerging cell and gene based technologies over the years. Early work on skin substitutes, for example, laid the foundation for the tissue engineering field by relying on biomaterials to create the right milieu to allow tissue-like organization of seeded (*ex vivo*) or invading (*in vivo*) cells (Bell et al., 1979; Yannas, 1992), while separate efforts were being undertaken to devise ingenious ways to transfer foreign genes into tissues by using projectiles to penetrate the skin (Williams et al., 1991). The amalgamation of separate approaches allowed biomaterials to support tissue organization *ex vivo* and to implement new gene transfer techniques, resulting in *ex vivo* construction of devices from gene-modified cells for transplantation (Tai and Sun, 1993). From these beginnings, biomaterials have evolved to now enable several key technologies at the center of nucleic acid-based therapies. Below we summarize the impact of biomaterials in key areas important for the future of nucleic acid therapeutics.

### More Intelligent NPs (Figure 2)

Increasing complexity (i.e., functionalization) in nucleic acid bearing NPs will be the way forward to realize more effective therapeutic outcomes from nucleic acids. Despite emergence of a wide range of synthetic, “intelligent” materials for NP fabrication in the last decade, there is still a need to create new functional NPs for hard-to-transfect cell types. Using commercial and in-house developed non-viral reagents, the authors frequently encounter cell types (e.g., leukemic stem cell lines and certain mesenchymal stem cells) that are exceptionally difficult to transfect. Patient-derived cells in particular have shown variable results in our hands for siRNA-mediated silencing of therapeutic targets, with significant fraction of cells either not responding or responding weakly to nucleic acid treatments (unpublished observations, and Gul-Uludag et al., 2014; Landry et al., 2016; Valencia-Serna et al., 2019). We recognize that increasing complexity in NP design, while improving performance, places extra burden on manufacturing processes, so that new design features amenable for scale-up will be especially critical for clinical translation.

**Figure 2:**
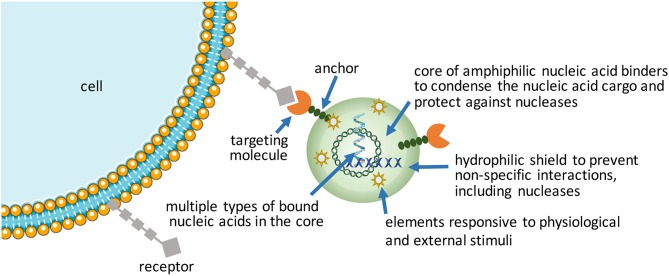
Design of intelligent NPs for delivery of nucleic acids.

Packaging nucleic acids with a combination of cationic and lipidic biomaterials have been recognized to improve delivery as compared to either moiety alone (Incani et al., 2010; Liu et al., 2010). Additional functionalization of NPs has been possible with antibodies (Kedmi et al., 2018) and other ligands (Guan et al., 2019) using lipidic anchors, and peptides/proteins by electrostatic anchors on self-assembled systems (Dong et al., 2018). Excessive cationic charge density, a recognized limitation of NPs, could be altered by incorporating anionic macromolecules into NPs. We incorporated anionic hyaluronic acid into NPs, either as a surface coating or additive into the core, that controlled the 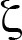-potential of NPs in a predictable way as well as increased the propensity of NPs for dissociation that was beneficial for both pDNA (Remant Bahadur et al., 2015) and siRNA delivery (Parmar et al., 2018). Another benefit was improved stability of NPs (Rose et al., 2013), with direct implications for *in vivo* administration. This benefit was not unique for multivalent polymeric carriers, but even liposomal systems such as the commercially available Fugene^TM^ which derived a beneficial effect from the additives in complexes (Nakamura et al., 2015); the additives in this case were hydrophilic/uncharged PEG and anionic tRNA that were widely different in molecular features, yet they were both able to enhance the transcriptional activity of a minimal PCR-amplified DNA expression cassette in the robust HEK293 cells (Nakamura et al., 2015). Different mechanisms might therefore be responsible (or effective) to weaken nucleic acid binding just enough to enhance the availability of nucleic acids intracellularly. Alternatively, the cationic charge density initially required for nucleic acid complexation could be removed by a controlled chemical cleavage (Jiang et al., 2019), while the NPs are retained in place by covalent linkages or possibly by other affinity interactions such as the hydrophobic domains. Improved toxicity was reported against a well-recognized liposomal formulation as a result of charge reduction, but systematic studies on the beneficial effect of reducing cationic charge density remains to be reported (Jiang et al., 2019).

Self-assembly has been favored in the hands of most researchers due to its convenience to create NPs at the time and site of application, in addition to the possibility of seamlessly incorporating additional functional molecules into the NPs. However, pre-manufactured NPs that bear nucleic acids may reduce variability associated with “on-the-spot” NP preparations and improve stability during the delivery. Nanogels, physically or chemically crosslinked polymeric networks with high water content, are emerging as leading candidates in this regard (Zilkowski et al., 2019). Nanogels with targeting ligands can entrap nucleic acids by electrostatic interactions or “irreversible” covalent linkages. Cargo can be loaded during synthesis or post-synthesis. Compared to conventional hydrolytically-degrading NPs, nanogels offer the possibility of more robust degradation under defined redox, pH and microenvironmental conditions, leaving behind a smaller footprint. To create a biomimetic means to shield the excess cationic charge of nanogels, they have been decorated with “recognizable” polysaccharide chains in a way replacing the synthetic PEG decoration. Polysaccharide chains can undergo degradation at sites of specific enzymatic activity (Nishimura et al., 2017), so that cellular uptake is facilitated at these sites, preventing non-specific interactions caused by the cationic charge in other (especially serum) sites. Recently, NPs prepared with adenosine-5′-triphosphate (ATP) responsive phenylboronic acid (PBA) bearing polymers (Naito et al., 2012) or ATP-responsive aptamers (Mo et al., 2015) are providing new ways of releasing the cargo intracellularly in response to high cellular ATP concentration that is typically absent in the extracellular space. The ATP-triggered release is reminiscent of the glutathione (GSH)-sensitive disulfide linkages, an earlier approach for intracellular cargo release triggered by the severe GSH gradient between the intracellular and extracellular compartments. The latter approach seems simpler to implement but the relative efficiency of intracellular vs. extracellular cleavage rates under physiological conditions for the two approaches remains to be thoroughly compared. Both of these approaches rely on physiological stimuli to execute the cargo release. If one wishes to rely on an external trigger for cargo release, analogous to inducer-activated gene expression or silencing, Khan et al. have recently reported an externally activated approach to nucleic acid release (Khan et al., 2017), whereby the small molecule tetrazine was capable of breaking the trans-cyclooctene linkages holding onto siRNAs in a NP. The relative stability of the trans-cyclooctene linkage and its specificity to tetrazine cleavage was proposed as a superior ‘on-demand' release of nucleic acids, where the proof-of-principle studies were reported in cell culture conditions.

Finally, another approach to intelligent NPs proposed by Mirkin group is to create spherical nucleic acids (SNAs) assembled on NP cores; they were shown to effectively penetrate the blood-brain-barrier as well as the blood-tumor-barrier and implement the RNAi silencing pathway (Cutler et al., 2012; Young et al., 2012; Jensen et al., 2013; Li et al., 2018). This seems to be possible with clustering of polynucleotides (which by themselves do not effectively cross cell membrane), perhaps due to an increased fluid phase uptake of the NP configuration and/or the lipophilic NP core.

### mRNA Delivery to Replace pDNA Therapy

mRNA delivery has been pursued for some time now with successful mRNA transfer by lipidic carriers reported as early as 1989 (Malone et al., 1989). Recent efforts to modify the nucleic acid for improved stability, better translation and lower immunogenicity are opening up new possibilities for its broader deployment (Kormann et al., 2011). Given the wealth of already developed carriers for other types of nucleic acids, a critical issue is whether we need new carriers for mRNA delivery or are the previous carriers sufficient to deploy this particular nucleic acid. While debatable, new carriers that rely on charge alteration to reduce/eliminate the electrostatic binding to mRNA and making mRNA freely available to translation machinery have been reported (McKinlay et al., 2017). Even with these apparently effective carriers, the outcome from *in vivo* mRNA administration is short-lived, with expression levels returning to baseline levels within ~48 h time frame. Some studies indicate that carriers previously developed for other nucleic acids can be employed, and in head-to-head comparisons, optimized pDNA delivery could be even superior to mRNA delivery in some cases, for example with human bronchial epithelial cells and lung delivery *in vivo* (Guan et al., 2019). Other studies reported the opposite; when comparing mRNA vs. pDNA delivery, biomaterial scaffolds were reported to display superior mRNA-induced transgene expression for a longer duration *in vitro* (Elangovan et al., 2015; Balmayor et al., 2016). The nature of the delivered gene and its regulation, the nature of the carrier (i.e., its influence on intracellular pharmacokinetics of the cargo) as well as the specific cellular system (i.e., in particular endocytosis efficiency against different cargos and ability for nuclear import) could be the reason(s) for the observed differences. It is likely that minicircle pDNA (that bear no non-essential genetic elements) with improved design over the traditional pDNA could be superior over the mRNA based gene expression, while mRNA could be superior over the traditional pDNAs. On the other hand, optimization of terminal repeats and/or incorporated modified bases make significant differences in mRNA performance, so that the effectiveness of mRNA over conventional pDNAs may be variable in different systems and this may take some time to clarify. Our own experience indicates that relative performance of pDNA vs. mRNA is cell-line dependent, and that some cells display better transgene expression from mRNA polyplexes, while others provide more robust expression from pDNA (unpublished). This observation possibly reflects the nuclear import capability of the cells, their proliferation rate and/or the capability of the carrier to deliver the pDNA to the nucleus.

Vaccination seems to be an especially appropriate area for mRNA administration where the adjuvant ability of mRNA may be additionally beneficial for a strong transient response. Implants where the biomaterials act as a local matrix (scaffold) to modulate the release of mRNA are an effective approach to vaccination (Chen et al., 2018), especially if prolonged local presence and/or controlled release is optimal. Scaffolds could be viewed as passive carriers of mRNA particles; transfection reagents are usually designed to transfect cells with no specific consideration to scaffolds (Steinle et al., 2018). The avidity (i.e., overall strength) between the nucleic acid and the complexing biomaterial has been shown to control mRNA release from NP formulations (Lallana et al., 2017) and it is likely that such a relationship will hold true for macroscopic scaffolds as well. In a presumably continuous scaffold, this will require control over the density of charges if no other “binders” are considered. The relatively weak immune-adjuvant features of mRNA could be further improved by employing double stranded mRNAs that are highly recognized by pattern recognition receptors (PRRs); in this case, an optimal length of double strands was needed to balance the immunostimulation with translational activity (Uchida et al., 2018). Alternatively, polymer-condensed mRNA could be entrapped in lipidic envelopes to enhance uptake and adjuvant activity (Persano et al., 2017).

Bone induction by mRNA translation is another indication where transient transgene expression might be sufficient for clinical success. Morphogens such as Bone Morphogenetic Proteins (BMPs) are known to “kick-start” the osteogenesis process beyond a critical concentration and their continued presence might not be required to sustain tissue induction and repair. The precise design of mRNA with particular chemical and end-group modifications are critical for effective translation, but several successful configurations have emerged for relatively long term protein production indicating some flexibility in the mRNA design. A longer sustained expression was noted when a BMP-2 morphogen was delivered with mRNA in scaffolds, presumably reflecting favorable pharmacokinetics and cell exposure (i.e., gradual vs. bolus) (Balmayor et al., 2017; Guan et al., 2019). Recent studies led by Balmayor et al. (2017), Badieyan et al. (2016) and Zhang et al. (2019) employed small animal models to assess the potential of mRNA-based bone repair, with so called “transcript activated matrices” (TAMs). A range of cells including easy-to-transfect cell lines and primary cells derived from adipose tissue and bone marrow were effectively induced for mRNA translation and significant secretion of therapeutic proteins (Badieyan et al., 2016; Balmayor et al., 2017; Zhang et al., 2019). It appears that robust effects were obtained even though the scaffolds were not optimized for bone repair (Elangovan et al., 2015). The elimination of the additional nuclear import barrier in primary cells, which is the limiting step for pDNA delivery, is an important advantage for deploying mRNA and makes this nucleic acid the preferred agent for delivery. Older studies, however, also showed some bone repair with pDNA based systems (so called “gene activated matrices,” GAMs) in similar preclinical models (Ono et al., 2004; Huang et al., 2005; Zhang et al., 2009; Qiao et al., 2013). Limited regeneration was noted in early investigations with BMP-4 pDNA/PEI25 (25 kDa branched PEI) implants around defect edges (Huang et al., 2005) potentially due to toxicities of high pDNA/PEI25 dose (200 μg of pDNA and likely >200 μg of PEI25) (Plonka et al., 2017; Khorsand et al., 2019). Ono et al. employed a hydroxyapatite scaffold to deliver cationic liposome condensed pDNA in a rabbit cranial defect, where the BMP-2 pDNA induced new bone tissue had penetrated halfway into the defect after 9 weeks (Ono et al., 2004). Qiao *et al*. employed PLGA particles containing BMP-2 pDNA/PEI25 and gelatin sponges in a calvarial defect model; bone formation was stimulated by BMP-2 gene delivery at defect edges (Qiao et al., 2013). More recently, in a head-to-head comparison, a GAM with pDNA and a TAM with mRNA for BMP-9 expression were found to be equivalent for bone induction *in vivo* (Khorsand et al., 2019), again suggesting no clear impediment to pDNA based GAMs in tissue induction. While difficult to compare these independent studies, the authors believe that mRNA may provide more robust osteogenic transformation *in vivo* (given the lower dose of mRNA in implants vs. pDNA), leading to comparatively better results in certain animal models. Lower doses of nucleic acids/synthetic carriers may minimize adverse inflammatory/immune reactions that may impede new tissue induction. The delivery system used in mRNA delivery were not particularly tailored in these early studies (i.e., PEI and a cationic lipid), so that improved carriers are bound to further improve regeneration with reduced doses. Collectively, these studies indicate that a robust translation of mRNA for ~10 days *in vitro* appears to be sufficient for effective tissue induction in small animal models. Investigations in larger animals, however, will be required to truly assess clinical potential. Considering that μg quantities of BMP proteins are needed for effective regeneration in small animals and that clinical therapy in the past relied on 10–20 mg of the protein *in situ*, it will be important to determine the functional mRNA doses in larger preclinical models to better assess its potential for clinical translation.

### Long-Acting Gene Expression With Non-viral Systems

The emerging T-cell therapy has again shined a light on the need for long-term gene expression with non-viral approaches. Transposons have emerged as a viable alternative to integrating viruses to this end whose utility is now being tested in clinical studies (Kebriaei et al., 2016; Tipanee et al., 2017b). The SB system relies on integration-enabling transposases, which can be delivered in protein form, in an expression plasmid, including the minicircle approach (Holstein et al., 2018) or more recently with mRNA (Monjezi et al., 2017). The latter approach obviates the need for nuclear delivery and may be a superior alternative due to transient induction of a transposase that will limit long-term transposition and hence unpredictable events. The current process of transposon delivery operates with nucleofection, which is a special form of electroporation with “facilitating” buffers. Although effective, nucleofection process is associated with loss of viability in a significant proportion of treated cells, so that it hampers *ex vivo* expansion efforts and prolongs attainment of critical mass of cells needed for transplantation. With an optimized combination of expression/integration system, 25–35% of cells were shown to retain the transgene expression in hard to transfect CD34+ hematopoietic stem and progenitor cells (HSPCs) (Holstein et al., 2018), based on the assessment of transgene expression in HSPC colonies or vector copy integrated/diploid genome. From a safety perspective, integration profiles of the transgene were favorable for the SB system over lentiviral vectors in human HSPCs, leading to random integration away from transcriptional regulatory elements of active genes and other “hotspots.” However, with integrating vectors, a finite risk of long-term adverse effects is present and should be considered in the face of benefit to be derived (Moffett et al., 2018).

Implementing transposon-based long-term gene expression *in vivo* will be desirable but also particularly challenging. Toward this goal, a NP system was described that were deigned to transfect T-cells (functionalized with an anti-CD3e f(ab')2 fragment) in a murine model (Smith et al., 2017b); *in vitro* results indicated a relatively low level of transfection (~4% of population), but this was sufficient for target cell killing and matched the performance of lentiviral-modified T-cells. The low levels of transfection will translate into benefit in terms of lower non-specific binding (and modification) of non-target cells. The extent of *in vivo* modification was similar with ~5% of circulating T-cells displaying transgene expression after 6 days, but the cells expanded with increasing population of cells displaying CAR expression (~20% after day 12), that was dependent on transposase delivery. In the absence of transposase delivery, no effective anti-tumor response was seen, clearly indicating the beneficial effect of vector integration. A similar delivery system was used for transiently transfecting T-cells with mRNA, whose biocompatibility was compared to electroporation modified cells (Weiden et al., 2018a); the modification with the non-viral system was implemented with lower adverse effects on cells, as evident in subsequent expansion rate *ex vivo*. It was interesting to note that this study also used a transiently expressed transcription factor Foxo1 (from mRNA whose expression lasted for ~5 days) that favors the expansion of desirable population of T-cells, that may provide a superior alternative to transient delivery systems towards the ultimate goal of integrated (long-acting) vectors (Broderick and Humeau, 2017).

### Expanding Genetically Modified Cells

**Figure 3:**
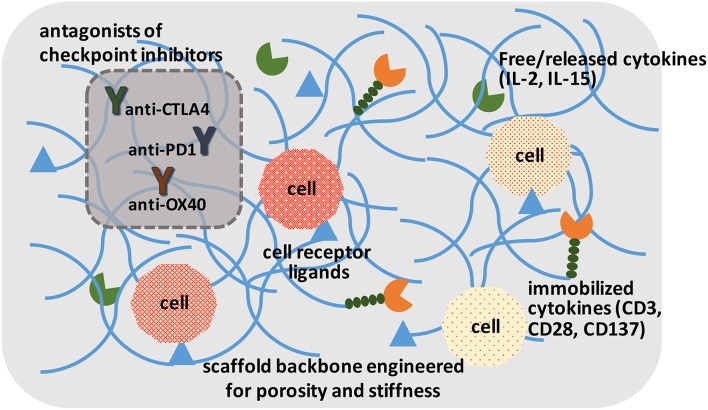
A schematic of ideal scaffolds to expand and/or activate T-cells for disease management. A sophisticated scaffold could be designed to support cell survival and expansion based on cell-attachment ligands, free/released cytokines, and immobilized ligands to promote cell proliferation. The cells could be activated with local presentation of antagonists of checkpoint inhibitors. The scaffolds could serve for *ex vivo* expansion of T-cells, as well as *in vivo* activation of T-cells.

Irrespective of the modification approach, CAR and other genetically modified cells may need to be expanded to provide them with a survival advantage when grafted into a host. This has been implemented in the past by using soluble cytokines, intracellular expressed factors (Weiden et al., 2018a) and immobilized ligands on tissue culture surfaces. The “Dynabead” system with immobilized CD3/CD28 antibodies on microparticles has been commercialized towards this end. Alternatively, one can employ biomaterials scaffold-conjugated ligands to enhance stimulation over that of soluble cytokines, and avoid additional manipulation of cells for transcription factor expression. Hydrogels derivatized with α_2_β_1_ collagen receptor binding GFOGER peptide or multiple integrin-binding RGD motif have been described that support T-cell expansion *in vitro* and housing after grafting the cells *in vivo* (Cheung et al., 2018; Weiden et al., 2018b). The hydrogels could immobilize ligands or provide local release of cytokines important for cell expansion (Figure 3), which may be difficult to implement with systemically administered agents. Infiltrating cells can be stimulated and expanded within designer niches (Ren and Lim, 2018). Delivering anti-CTLA-4 and anti-OX40 mAbs has been described to stimulate tumor-infiltrating killer T-cells with scaffolds in the vicinity of resected tumors (Wang et al., 2016). It has been possible to create scaffolds from nucleic acids (DNA-based) to release immune stimulatory PD-1 blocking agents (Lynn et al., 2019). The importance of size, architecture and ligand density, among others, are beginning to be elucidated for *in vivo* expansion of T-cells (Liu et al., 2018), while a similar approach is implemented for *ex vivo* expansion. It has been recognized for some time that immobilized antibodies are more potent in stimulating T-cell expansion compared to soluble ligands, and a mechanosensor receptor (Piezo1) was recently identified as a mediator of TCR activation (Zhang et al., 2018). This provides a mechanistic link on how mechanical properties of a scaffold could affect T-cell stimulation and expansion directly, and may provide a more rational design of the biomaterials scaffold to optimize TCR activation and T-cell expansion.

Vaccination with pDNA is continuing to be explored with biomaterial-based delivery and adjuvant systems (Zhang et al., 2018), with muscle and skin sites (by electroporation) popularly used for *in situ* expression of tumor antigens from pDNA directly (Amante et al., 2015; Broderick and Humeau, 2017). Inhibition of immune checkpoints CTLA-4 and PD-1 is making inroads to enhance the anti-tumor response with *in situ* expressed tumor antigens (Lopes et al., 2018). Simple injection of pDNA without the use of electroporation has been made effective with the use of a combination of cationic lipid formulations, where the 2-dis-tearoyl-sn-glycero-3-phosphoethanolamine-N-[methoxy(PEG-2000)] was critical in supporting expression of antigens and long-term antibody response (Ho et al., 2018). Hydrolytically-cleaving polyesters have also been shown to successfully elicit effective antibody response against pDNA-coded antigens, with a lipid-modifed PEI (PEI1.8-deoxycholic acid) facilitating local transfection and antigen expression (Giang Phan et al., 2019).

## Concluding Remarks on Future Nucleic Acid Therapies

Synthetic, precisely engineered biomaterials and self-assembled systems from such biomaterials are leading the way to enable a diverse array of therapeutic modalities that rely on nucleic acids. The prospect of improved clinical safety of the biomaterial-based delivery is driving this endeavor and significant efforts are in place now to enhance the effectiveness of the delivery, while allowing a high degree of modification of “hard-to-transfect” cells and realizing permanent modification (whether it may be transgene expression or silencing). Nucleic acids themselves derived from DNA and RNA molecules have the potential to replace synthetic biomaterials and act as carriers for nucleic acid agents (Hu et al., 2018). It has been possible to create responsive systems to release different effector molecules from a scaffold of nucleic acids with precise controlled features. One can envision delivering CpG oligodeoxynucleotides, that bind TLR9, from DNA scaffolds effortlessly to stimulate dendritic cells against tumors (Bourquin et al., 2008). The possibilities are diverse, but whether they can be produced in economical terms, their *in vivo* stability be controlled and adverse reactions *in situ* be minimized remains to be seen for such nucleic acid scaffolds. While delivery with biomaterials for therapeutic purposes has been the main focus, one can envision relying on nucleic acids for “preventative” medicine as well; with the identification of aberrant genes and/or miRs before manifestation of clinical symptoms, one has the opportunity to employ nucleic acids before disease development. One can envision deleting “aberrant” cells or restoring normal physiology ahead of detectable symptoms. Perhaps our next generation of “vitamins” will be based on nucleic acids as preventative remedies; nevertheless, the functional use of nucleic acids will rely on designer biomaterials and nano-engineered systems in order to present the nucleic acids to the appropriate cells in the appropriate manner.

## Author Contributions

All authors contributed to the conceptualization, literature search/review, and writing of the article. Final editing was undertaken by HU.

### Conflict of Interest Statement

HU is a founder and share holder in a private company (RJH Biosciences Inc.) intended to develop nucleic acid based therapies and declares conflict of interest. The remaining authors declare that the research was conducted in the absence of any commercial or financial relationships that could be construed as a potential conflict of interest.

## Footnotes

^1^FDA letter to Sarepta Therapeutics Inc. on eteplirsen approval. Available online at https://www.accessdata.fda.gov/drugsatfda_docs/nda/2016/206488_summary%20review_Redacted.pdf

^2^Yescarta™ (axicabtagene ciloleucel) suspension for intravenous infusion Initial U.S. Approval: 2017. Available online at: https://www.fda.gov/files/vaccines%2C%20blood%20%26%20biologics/published/Package-Insert—YESCARTA.pdf

^3^Kymriah™ *(tisagenlecleucel), first-in-class CAR-T therapy from Novartis, receives second FDA approval to treat appropriate r/r patients with large B-cell lymphoma (2018)*. Available online at: https://www.novartis.com/news/media-releases/kymriahr-tisagenlecleucel-first-class-car-t-therapy-from-novartis-receives-second-fda-approval-treat-appropriate-rr-patients-large-b-cell-lymphoma

## Abbreviations

DNA: deoxyribonucleic acid
RNA: ribonucleic acid
siRNA: small interfering RNA
shRNA: short hairpin RNA
mRNA: messenger RNA
miR: microRNA
pDNA: plasmid DNA
SB: sleeping beauty
TIR: Terminal Inverted Repeat
ASO: Antisense oligonucleotide
RNAi: RNA interference
lncRNA: Long non-coding RNAs
circRNA: circular RNA
gRNA: guide RNA
PEG: polyethyleneglycol
PEI: polyethyleneimine
mAb: monoclonal antibody
CAR: Chimeric Antigen Receptor
APC: Antigen presenting cell
GAM: Gene Activated Matrix
TAM: Transcript Activated Matrix
TCR: T-cell Receptor
NP: nanoparticle
PLGA: polylactide-co-glycolide
CRISPR: Clustered Regularly Interspaced Short Palindromic Repeat.
